# Identifying Cell Class Specific Losses from Serially Generated Electroretinogram Components

**DOI:** 10.1155/2013/796362

**Published:** 2013-09-09

**Authors:** Christine T. O. Nguyen, Algis J. Vingrys, Vickie H. Y. Wong, Bang V. Bui

**Affiliations:** Department of Optometry and Vision Sciences, The University of Melbourne, Parkville, VIC 3010, Australia

## Abstract

*Purpose*. Processing of information through the cellular layers of the retina occurs in a serial manner. In the electroretinogram (ERG), this complicates interpretation of inner retinal changes as dysfunction may arise from “upstream” neurons or may indicate a direct loss to that neural generator. We propose an approach that addresses this issue by defining ERG gain relationships. *Methods*. Regression analyses between two serial ERG parameters in a control cohort of rats are used to define gain relationships. These gains are then applied to two models of retinal disease. *Results*. The PIII_amp_ to PII_amp_ gain is unity whereas the PII_amp_ to pSTR_amp_ and PII_amp_ to nSTR_amp_ gains are greater than unity, indicating “amplification” (*P* < 0.05). Timing relationships show amplification between PIII_it_ to PII_it_ and compression for PII_it_ to pSTR_it_ and PII_it_ to nSTR_it_, (*P* < 0.05). Application of these gains to *ω*-3-deficiency indicates that all timing changes are downstream of photoreceptor changes, but a direct pSTR amplitude loss occurs (*P* < 0.05). Application to diabetes indicates widespread inner retinal dysfunction which cannot be attributed to outer retinal changes (*P* < 0.05). *Conclusions*. This simple approach aids in the interpretation of inner retinal ERG changes by taking into account gain characteristics found between successive ERG components of normal animals.

## 1. Introduction

**Figure 1:**
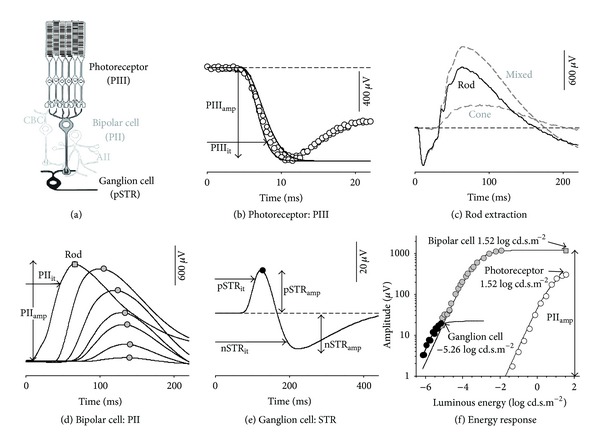
ERG analysis: saturated ERG components arising from the rod through pathway. (a) Schematic of retinal cytoarchitecture showing rod photoreceptors, bipolar, and ganglion cells with relevant interneurons (AII amacrine and cone bipolar (CBC)). (b) a-wave was modelled with a delayed Gaussian over an ensemble of two luminous energies (1.22 and 1.52 log cd·s·m^−2^) to derive Rm_PIII_ (PIII_amp_). The time to reach 80% trough amplitude was taken as the implicit time (PIII_it_). (c) To isolate rod responses, a paired flash paradigm was implemented, and the rod waveform was derived following subtraction of cone from mixed waveforms. (d) Rod PII waveform was derived by subtracting the modelled PIII (Panel (b)) from rod isolated waveforms (Panel (c)). Rod PII implicit time was taken at 80% of maximal amplitude (PII_it_). (e) STR amplitude was measured at the peak (pSTR_amp_) and trough (nSTR_amp_). STR implicit time was taken at 80% of maximal amplitude (pSTR_it_, nSTR_it_). (f) Analysis undertaken on components at their saturated response. Energy-response functions illustrate that, for photoreceptor (PIII, white circles) and bipolar cell (PII, grey circles), this occurs at 1.52 log cd·s·m^−2^ and for ganglion cell (pSTR, black circles) at −5.26 log cd·s·m^−2^.

The electroretinogram (ERG) has been utilized as a measure of retinal function for over 100 years and reflects the massed retinal response following stimulation by light. One of the advantages of using the ERG is that specific components of the waveform can be attributed to particular cellular generators in the retina [1]. The retina is a serial processor where cells are arranged into the through or lateral pathways [2]. In simplistic terms (see Figure 1(a)), in the through pathway, the photoreceptors (first-order neuron) convert light into a transmembrane potential which is transmitted to bipolar cells (second-order neuron) at their synaptic terminals. Subsequently, the bipolar cells feed this information onto the output cells of the eye, the ganglion cells (third-order neuron).

These three key retinal cell classes of the through pathway have been shown to contribute to particular components of the ERG waveform (see Frishman [3] and Weymouth and Vingrys [4] for review). The early portion of the ERG's a-wave (PIII) has been shown to be largely photoreceptoral in origin [1, 5–8]; the b-wave (PII) is thought to be generated by bipolar cells [9–13], and in rodents the scotopic threshold response (STR) has proximal neuronal generators involving ganglion and amacrine cells [14–16]. As extraction of PIII and PII components have been shown to be a more accurate reflection of photoreceptoral and bipolar cell activity than are the a- and b-wave, the former terminology will be utilized in this paper.

However, as the processing of the retina (and in turn the ERG) occurs in a serial manner this means that outer retinal defects will have a knock-on downstream effect to inner retinal neurons. More specifically, dysfunctional outer retinal neurons (first- or second-order neurons) will reduce their input to inner retinal neurons and decrease inner neuronal activity (second- or third-order neurons resp.). Thus a loss of ERG components arising from bipolar or ganglion cells may reflect an upstream dysfunction or an injury to the cell itself. This concept is wellrecognised in the ERG literature between the first- and second-order neurons, and in human studies the ERG processing or “gain” relationship between photoreceptors and bipolar cells is well defined [17, 18]. What is unknown is whether this same gain relationship manifests in the rat ERG and moreover whether the same relationship occurs from bipolar to ganglion cells. Previous attempts that have been made to address this issue will be considered next. 

Expressing the PII_amp_ as a ratio of the PIII_amp_ (analogous to the b-wave/a-wave ratio) [19] is one common method used to identify PII specific deficits. Treatments that cause no alteration to this ratio (PII_amp_/PIII_amp_) imply that any bipolar cell changes are downstream expressions of photoreceptor changes. A reduction in the PII_amp_/PIII_amp_  ratio would indicate a specific loss at the bipolar cell level. Another approach that can be used for this purpose is to normalise parameters to control and consider the percentage change in an attempt to differentiate direct and downstream mechanisms [20, 21]. This method expresses a treatment effect as a percentage of the control. Using this approach, a PII percentage loss larger than of PIII would indicate a direct effect at the bipolar cell. The ratio analysis and percentage change approach are robust in cases where the gain relationship between two cell-class responses is unity; that is, a PIII loss will lead to an equivalent PII loss; however, it fails if the gain relationship between cells differs from unity. What is needed is a better understanding of the “gains” between successive ERG waveform stages or components to see if such approaches are justified. We will define these in this study.

Another approach utilised in the literature is to consider sequential processing losses by adopting a computational waveform model [17]. This method considers the influence that altering one part of the waveform (i.e., negative deflection PIII) will have on another part of the same waveform (i.e., positive deflection PII). Although useful, this approach is only applicable to those ERG components that exist within the same waveform and excludes components measured at a different intensity or stimulus modality from the same eye. Consequently, it cannot be applied to different waveforms meaning that gain relationships to proximal cell classes cannot be assayed, that is, bipolar cell to ganglion cell gain, PII to pSTR. We will attempt to address this issue by proposing a novel method for analysis.

Thus, this study defines the ERG gain relationships between cells within the rod-through pathway, including photoreceptors, bipolar cells, and ganglion cells. If the gains at different stages of the retina are unity, then loss will transfer from one step to another unaltered. However, in the presence of nonunity gain, amplification or compression of loss can occur. Although a unity gain has been shown for humans between PIII and PII amplitudes [17, 18], it is not known if this is the case for rodent ERGs. Furthermore, it is yet to be determined whether the presumption of unity gain applies proximal to bipolar cells. At the ganglion cell level another layer of complexity in processing exists, including lateral communication, inhibitory feedback [22–24], and further convergence of signals [2, 25]. Thus, unity gain between outer retinal ERG components and the STR appears unlikely. Moreover, as these ERG components do not express in a common waveform, a computational waveform model cannot be applied to consider losses, and thus a new approach is needed for their evaluation.

The aim of this paper is to develop an easily applicable model to differentiate between deficits in inner retinal ERG components that arise from direct injury to inner retinal neurons or simply manifest as downstream expressions of outer retinal dysfunction. We propose that understanding the gain between successive stages can help in such evaluation when particular constraints are taken into consideration. To identify this gain we chose waveform components known to have contributions from elements in the through pathway and consider the inherent variability found across a cohort of “normal” animals. We reason that animals having a high photoreceptor output should produce a large PIII (a-wave), and this should result in a large PII (b-wave). Similarly, this large PII should affect lateral and third-order neurons and result in a larger STR. By considering the variability seen within a normal group, we should be able to define the “gain” between these waveform generators. 

To assay the robustness of our gain analysis we apply the technique to two rat models of retinal disease, one conventionally thought to preferentially alter photoreceptoral responses (*ω*-3 deficiency [19]) and the other believed to exert its effects throughout the retinal layers (diabetes [26]). For comparative purposes, we also apply current methods used in the literature and show how these may fail to define site-specific losses.

## 2. Materials and Methods

All experimental procedures conform to the Association for Vision in Research and Ophthalmology statement on the use of animals for scientific purposes. 

### 2.1. ERG Waveform Collection

Sprague Dawley rats (*Rattus norvegicus*) were housed as described previously [27]. For ERG measurements, ketamine : xylazine anaesthesia was used (60 : 5 mg/kg intramuscularly; Ketamil 100 mg/ml, Xylazil 100 mg/ml, Troy laboratories, Smithfield, NSW, Aust). Topical proxymetacaine hydrochloride (Ophthetic 5 mg/ml, Allergan, Frenchs Forest, NSW, Aust), 0.5% tropicamide (Mydriacyl 5 mg/ml, Alcon laboratories, Frenchs Forest, NSW, Aust) and 1.0% carboxymethylcellulose sodium (Celluvisc; Allergan, Irvine, CA) provided corneal anaesthesia, mydriasis, and hydration, respectively. Body temperature was maintained at 37 ± 0.5°C by a water heat pad. ERGs were recorded with custom-made cholrided silver electrodes with an active corneal electrode and a scleral reference. A stainless steel ground (F-E2-30 Grass Telefactor, West Warwick, RI) was inserted subcutaneously in the tail. Stimuli were brief white flashes (0.1–1 ms, 5-W white LEDs, 5500°K; Luxeon Calgary, Alberta, Canada) delivered by a Ganzfeld integrating sphere (Photometric Solutions International, Huntingdale, Victoria, Australia) that had flash energy calibrated by an IL 1700 photometer (International Light, Newburyport, MA, USA). Luminous energies were collected over ~8 log units (−5.95 to 1.52 log cd·s·m^−2^) to be able to sample the various waveform components that reflect the through pathway neuronal generators. Further details regarding electrode configuration and flash characteristics are described by Nguyen et al. [28] and He at el. [29].

### 2.2. Gain Analysis

The input-output analysis, or gain, is applied to the cellular generators of the rod-through pathway as their components can be readily isolated in the rat ERG. These include rod-derived photoreceptor (PIII), ON-bipolar cell (PII), and ganglion cell (scotopic threshold response, STR) components (Figure 1(a)). The gain relationships are best considered between ERG components whose neural generators are serial. The relationship between PIII and PII will be considered, as rod photoreceptors communicate directly to ON-bipolar cells. As ganglion and amacrine cells receive input from bipolar cells (ganglion cells via AII amacrine cells in the rod pathway [2]), the PII to STR relationships will also be considered. Note that these signals can manifest in different waveforms (PII and STR, albeit both are at their saturated response), and we will propose a method that deals with this fact.

### 2.3. Requirements of ERG Gain Analysis

Multiple factors can alter the gain relationship between these ERG components so we reason that two requirements must be fulfilled when collecting and analysing the relevant ERG data. 

#### 2.3.1. Saturated ERG Amplitudes Are Used

Studies have shown that communication characteristics can change if components are not at their maximal output [13, 30, 31]. Consequently, ERG components need to be analysed at their saturated amplitudes, and the gain relationships derived apply only under these conditions. 

#### 2.3.2. Constant Adaptation Is Required

Single cell, eye cup preparations, and ERG studies have shown that synaptic gain varies with light adaptation [32–40]. Thus, ERG components must be compared under the same adaptation conditions, and in this study we consider the gain relationship of rod-isolated ERG components assayed under dark adapted conditions. 

Thus, by taking care to isolate relevant ERG components we hope to identify their putative neuronal generators and disentangle the influence of differing parts of the ERG waveform from each other. Although the computational waveform model [17] is designed to deal with the complexity of shifting components in a composite waveform, this approach is complicated. In this study particular care is taken to isolate the discrete neural generators of ERG components and in this manner simplify gain analysis. The next section will consider how this can be achieved. 

### 2.4. Defining ERG Components

#### 2.4.1. Rod Photoreceptor Response: Fast-PIII Component

 As the dark-adapted PIII in a rodent is dominated by the rod response, the mixed PIII can be used as a surrogate for the rod PIII. More specifically, pilot data (*n* = 21, data not shown) shows that cone responses make a 4.3 ± 0.4% contribution to the amplitude of the mixed a-wave at 1.52 log cd·s·m^−2^. This contribution is consistent with previous studies [4, 41] and within measurement noise for our system. Figure 1(f) (*n* = 8) illustrates that using a flash energy of 1.52 log cd·s·m^−2^ or more will yield a saturated rod photoreceptor response which is consistent with the literature [4].

 The rod photoreceptor response can be described by a model of phototransduction [6, 42–44] (Figure 1(b)). The model is optimised over an ensemble of ERG waveforms from two energy levels (Figure 1(b), 1.22 and 1.52 log cd·s·m^−2^, circles : data, lines : model). This model derives the saturated amplitude Rm_PIII_ (*μ*V), a sensitivity S (m^2^·cd^−1^·s^−3^), and a delay *t*_*d*_ (ms). Optimization was achieved by minimizing the sum-of-square (SS) merit function using the Solver module of an Excel spreadsheet (Microsoft). The Rm_PIII_ defined the saturated PIII amplitude (PIII_amp_) for our purposes. To account for potential changes in both *t*_*d*_  and S by the experimental manipulations, we calculated the implicit time required to achieve 80% maximum amplitude (PIII_it_, Figure 1(b)). Applying this 80% criterion for timing also limited intrusion of postreceptoral responses known to occur in the later part of the a-wave [42, 45]. It should be noted that rat rod a-waves will show saturation somewhere between 1.4 and 2.2 log cd·s·m^−2^ [4] and will grow in amplitude at higher energy levels due to intrusion from other processes [4].

#### 2.4.2. Rod Bipolar Cell Response: PII Component

The rod-isolated PII component is utilised as a measure of ON-bipolar cell response. Unlike the photoreceptor response the ERG b-wave contains substantial activity from rod and cone bipolar cells [4, 41]. To isolate rod-bipolar cell activity a paired flash was utilised [4, 26, 41, 46], consisting of a rod saturating flash (1.88 log cd·s·m^−2^) followed by a probe flash (1.52 log cd·s·m^−2^) with a delay of 500 ms to prevent rod intrusion (Figure 1(c)). The cone response obtained from the probe flash can be subtracted from the mixed waveform (Figure 1(c), grey dashed lines) to obtain a rod-isolated waveform (Figure 1(c), black line) [4, 26, 41]. The rod PII was then derived (Figure 1(d)) by subtracting the modelled PIII and low pass filtering (−3 dB at 46.9 Hz, Blackman window) to remove oscillatory potentials [4, 17]. 

To derive the rod saturated PII response, the rod PII peak amplitudes are modelled with a hyperbolic function from −4.8 log cd·s·m^−2^ [47]. As shown in Figure 1(f), this model is fit to rod-only data (i.e., below −2 log cd·s·m^−2^ and the paired flash rod response determined at 1.52 log cd·s·m^−2^, data: grey symbols, model: black line) and as such gives rod specific parameters (see logic detailed in [28]). This model derives a saturated amplitude (V_max⁡_, *μ*V), rod PII sensitivity (1/k, log cd·s·m^−2^), and a slope parameter (*n*) [48]. The V_max⁡_ parameter was used as a measure of saturated rod bipolar cell activity (PII_amp_).

The implicit time of the rod-isolated PII (PII_it_, Figure 1(d), 1.52 log cd·s·m^−2^) was taken as the time needed to reach 80% peak amplitude for consistency with the 80% PIII implicit time.

#### 2.4.3. Proximal Retinal Response: Scotopic Threshold Response

The ERG obtained with very dim light levels near absolute threshold [15] is known as the scotopic threshold response (STR) [49] and is believed to have major contributions from third-order neurons summating rod signals [3, 16, 50]. In rodents, ganglion cells have been shown to be responsible for generating the positive lobe of the STR (pSTR) and along with amacrine cells contributing to the nSTR. [14–16, 50, 51]. 

The distinction between the pSTR and PII components is illustrated in Figure 1(f) where at low intensities (black circles) there is a departure from the hyperbolic function that describes the rod PII energyresponse (solid line). This departure implies intrusion of a different mechanism consistent with the logic used to explain similar intrusions in behavioural energy-response data [52]. This particular ganglion cell response saturates at approximately −5.5 log cd·s·m^−2^ (Figure 1(f), black circles) consistent with previous studies [14, 16, 50]. 

To increase the signal-to-noise ratio of the STR, 20 responses were averaged (2-second interstimulus interval) and then low pass filtered (46.9 Hz, −3 dB, cosine transition). The saturated STR was assayed at −5.26 log cd·s·m^−2^ (Figure 1(f)) which is consistent with previous studies [14, 16]. The saturated pSTR amplitude (pSTR_amp_) was taken as baseline to peak, and the saturated nSTR amplitude (nSTR_amp_) was measured as baseline to trough (Figure 1(e)). Consistent with PIII and PII analyses, the implicit time was assayed using the time taken to reach 80% maximal amplitude (pSTR_it_, nSTR_it_, Figure 1(e)).

### 2.5. Determination of ERG Gains: Regression Analysis

**Figure 2:**
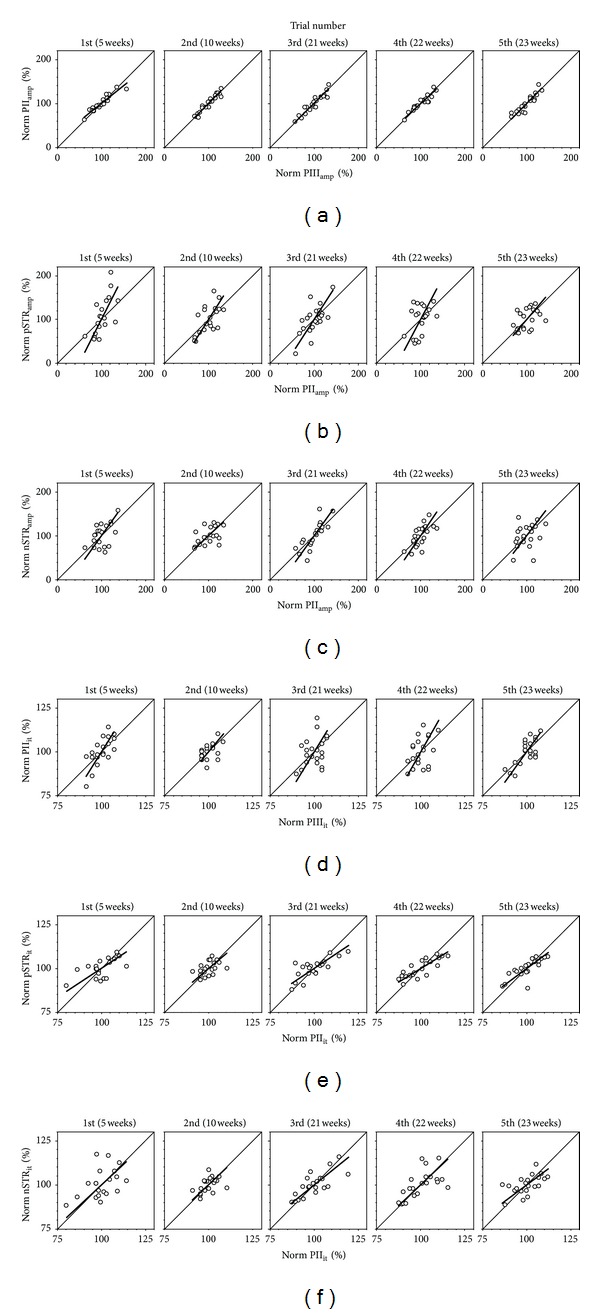
Normal gain between ERG components. The gain relationship between two ERG components is established by linear regression (thick line) of normalised upstream to the downstream ERG parameter for a group of control animals (unfilled circles) measured on 5 separate occasions. A slope of unity is represented by the thin diagonal lines. Statistics for this analysis are represented in Table 1. Correlations of (a) PIII_amp_ and PII_amp_ give slopes which are near unity. Correlations that produce slopes steeper than unity include (b) PII_amp_ and pSTR_amp_ (c)  PII_amp_ and nSTR_amp_, and (d)  PIII_it_  and PII_it_. Slopes shallower than unity were observed for correlations between (e) PII_it  _and pSTR_it_ and (f) PII_it_ and nSTR_it_.

When ERG components are extracted from a group of normal rats, one finds a significant amount of variability that can span ~±50% from the average of the population (see Figure 2). We posit that the variability within a “normal” control cohort can be utilized to expose the gain relationship between two ERG parameters. This is based on the presumption that these normal animals have a common gain between their components. This would mean that animals which exhibited a low amplitude for their ERG would consistently do so, yielding a relative stable gain at retest. To examine the repeatability of gains, a control cohort of rats had ERGs measured on 5 separate occasions as they aged (Figure 2). These were at 5, 10, 20, 21, and 22 weeks of age. Data from rats aged 20-22 weeks of age have been reported previously [28] to examine this control group against the effects of *ω*-3 deficiency (reanalysed here with permission). There were 13 rats common across the 5 to 23 weeks, and the total sample size for each time point varied between 20 and 21 animals.

Data are expressed normalized to the average of the relevant control group. This normalization is needed to allow comparison between the various ERG components, which differ greatly in amplitude and timing. In this manner, it can be determined whether an alteration in an upstream component will lead to a proportionate downstream change. Although some information may be obtained from examining the gain between raw amplitudes, such an analysis also contains bias towards vertically oriented extracellular currents. More specifically, photoreceptoral and bipolar cells produce vertically oriented extracellular currents, and thus PIII and PII components are large. In contrast the lateral-dominated extracellular currents produced by amacrine/ganglion cells are reflected in the smaller oscillatory potential and STR components. As such, normalisation is needed to facilitate comparison between these generators.

The gain relationship between the two components is described by a Deming regression (Figure 2, lines) which takes into account the data's variability in both *x* and *y* axes. The slope of the Deming regression indicates the gain characteristics where slope = 1 is unity gain, slope > 1 is referred to as “amplification”, and slope < 1 is referred to as “compression”. These slopes were averaged across the 5 trials and expressed as an average ± SEM, along with 95% confidence intervals (CI).

### 2.6. Application of ERG Gains to Retinal Disorders

In order to illustrate the application of our methodology, we applied the gains derived from normal animals to two treatments known to alter ERG outcomes. The first treatment (*ω*-3 deficiency) was chosen as it is well known to affect photoreceptoral function [19, 53–56], whereas the second treatment (diabetes) exhibits characteristic deficits in outer and inner retinal functions [26, 57–60].

To examine the effects of *ω*-3 dietary manipulation on the ERG, rodents were fed either *ω*-3 sufficient (*n* = 21) or deficient (*n* = 19) diets, 5 weeks before conception resulting in a 48.6% decrease in retinal docosahexaenoic acid (for further dietary details and tissue assays see Nguyen et al. [27, 28]). Detailed changes in ERG have been reported in Nguyen et al. [28], and, here, the gain analysis is applied to these animals (reanalysed with permission).

Diabetes was induced in a group of rats using tail vein injections of STZ (50 mg/kg *n* = 13). Control animals had tail vein injections of citrate buffer (*n* = 13). Diabetes was diagnosed based on physiological and biochemical parameters taken at 4, 8, and 12 weeks. These included elevated blood glucose levels (>15 mmol L^−1^), abnormal glycosylated hemoglobin (HBA1c > 7.0%), polyuria (>40 mL urine volume over 24 hrs), and polydypsia (>60 mL fluid intake over 24 hrs). Twelve weeks following treatment ERGs were collected. Data from these rats are reported in Bui et al. [57] and are reanalyzed here (with permission) utilising the ERG gain analysis. 

## 3. Results

### 3.1. Gain Relationships

 The amplitude gains derived from the 5 trials (Figures 2(a)–2(c)) are stable and reproducible. The PIII_amp_ to PII_amp_ gain is 0.95 and has 95% confidence limits encompassing unity (Table 1; 95% CI 0.73–1.07) indicating that a smaller PIII_amp_ results in a proportionately smaller  PII_amp_. In contrast, the PII_amp_ to pSTR_amp_ and PII_amp_ to nSTR_amp_  have gains that are significantly (*P* < 0.05) steeper than unity (Table 1(a); 1.64, 95% CI; 1.23–2.05 and 1.30, 95% CI: 1.03–1.56, resp.). Thus for a homogeneous photoreceptoral deficit (PIII) one can expect the same percentage PII loss but a relatively greater pSTR and nSTR reduction. In other words, a rod bipolar deficit will be amplified at the ganglion and/or amacrine cell level as measured using the STR.

In terms of timing (Figures 2(d)–2(f)), the 95% confidence limits indicate that the PIII_it_ to PII_it_ gain (1.54, 95% CI 1.19–1.88) was steeper than unity and that the PII_it_  to pSTR_it_ (0.71 95% CI; 0.56–0.85) and PII_it_ to nSTR_it_ (0.88, 95% CI; 0.78–0.97) gains were shallower than unity (Table 1(b)). Thus, a photoreceptor loss of sensitivity that expresses as an increased delay will be amplified to produce a later relative PII implicit time and contracted to yield relatively faster pSTR and nSTR implicit times compared to the PII. Our finding of nonunity gains means that amplitude or timing changes where these occur will lead to erroneous interpretations if considered as a ratio or percentage change. The following section will demonstrate the significance of this finding.

### 3.2. Application to Photoreceptor Dysfunction: *ω*-3 Deficiency

**Figure 3:**
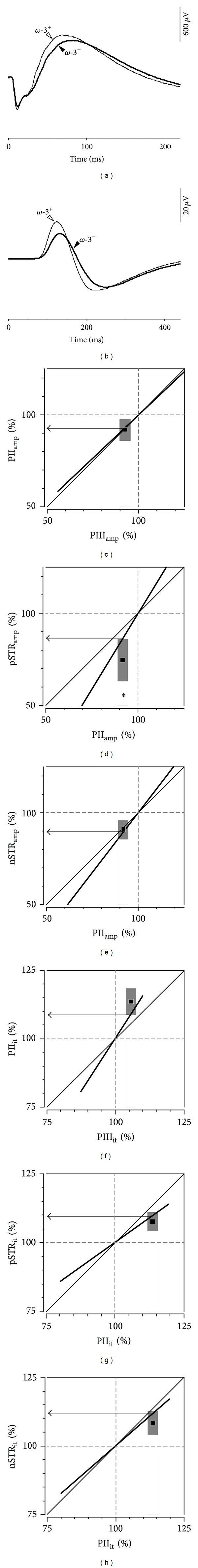
Application of ERG gain analysis in *ω*-3 deficiency. Averaged group ERG waveforms showing the effect of *ω*-3 dietary deficiency on the (a) rod-isolated a-b wave complex and (b) STR (reproduced with permission from Nguyen et al. [28]). In Panels (c)–(h) an ERG gain of unity is represented by the thin diagonal lines. The thick black lines represent the ERG gain determined in Figure 2 and Table 1. Grey bars represent the 95% confidence interval for the *ω*-3 deficiency treatment group. The horizontal arrows represent the predicted downstream loss given the measured upstream change. Asterisks (∗) indicate statistically significant direct loss at the respective component (*P* < 0.05). Thus *ω*-3 deficiency produces (c) PII amplitude reductions that can be expected from the PIII_amp_ decline, and (d) pSTR_amp_  losses are greater than predicted by ERG gain. The predicted pSTR_amp_ loss (arrow) falls outside the 95% confidence limits (grey bar) for the measured pSTR_amp_ loss in the treated group (filled square). (e) nSTR change can be accounted for by the reduction in PII_amp_. In terms of timing ((f), (g), and (h)) the predicted delays (arrows) fall within the 95% confidence interval of the treated groups. Thus the delays in the PII, pSTR, and nSTR can be accounted for by the delay in the outer retinal PIII.

Figure 3 illustrates the group average waveforms for a 1.52 log cd·s·m^−2^ flash that exposes the PIII and PII (Figure 3(a)) and a −5.26 log cd·s·m^−2^ flash that exposes the STR (Figure 3(b), *ω*-3 sufficient, thin line: *ω*-3 deficient, thick line). The change in PIII (−7.5 ± 3.6%) and PII (−8.2 ± 2.8%) amplitudes induced by the deficient diet is shown in Figure 3(c) (square symbol). The expected gain between these components derived from normal animals (diagonal thick line) predicts the magnitude of PII loss (arrow) given the PIII reduction. This means that the PII change can be completely accounted for by the diet-induced reduction in PIII input as the 95% confidence limits (−2.2% to −14.2%) for the measured PII change (grey bar) encompass the predicted loss. However, Figure 3(d) shows that the observed pSTR change is greater than what might be predicted from the PII change after allowing for a normal gain of 1.64 (arrow, −13.4%) as the 95% confidence limits of our data (−13.6% to −37.0) just fail to encompass this value. The predicted nSTR loss from a PII deficit −8.2% based on the gain for these waveforms (1.30, Figure 3(e) arrow) is −10.6%. The *ω*-3 deficient nSTR confidence limits (−3.7% to −14.7%) encompass this predicted loss. Thus the measured nSTR loss can be accounted for by normal gain processing between the PII and nSTR, which in turn can be attributed to the PIII amplitude deficit induced by *ω*-3 deficiency.

The measured PIII delay in implicit time in *ω*-3 deficient animals (+5.7 ± 1.6%) combined with the PIII_it_ to PII_it_ gain (1.47, Figure 3(f)) predicts a +8.8% PII delay (arrow). While the measured PII implicit time (+13.6 ± 2.3%) was relatively slower than that of the PIII (*P* < 0.001), its confidence limits (+8.7% to +18.5%) encompass the predicted value indicating that PII timing reflects serial processing of the slowed PIII. 

Although the pSTR implicit time delay of +7.6 ± 1.6% was statistically less than the PII delay (+13.6 ± 2.3%, F_3,18_ = 13.7, *P* < 0.001), its confidence limits (+4.2% to +11.0%, grey bars) encompass the +8.4% delay predicted from normal processing (arrow, Figure 3(g)). Likewise, the predicted nSTR implicit time delay (+11.9%, arrow) calculated from the measured PII delay (+8.2 ± 2.9%), taking into account normal gain (arrow, Figure 3(h)), falls within nSTR variability (95% confidence limits, +4.0% to +12.7%). Thus, the pSTR and nSTR implicit time delays reflect the PII delay, which in turn reflects the PIII delay induced by *ω*-3 deficiency. 

### 3.3. Comparison with Previous Analytical Methods: *ω*-3 Deficiency

For comparison, previous methodologies used in the literature including ratio and percentage change analyses are also applied to this data to contrast these methods. The computational waveform method is not applied to this study as it is only applicable to those components within the same waveform (i.e., photoreceptoral PIII and bipolar cell PII but not ganglion/amacrine cell STR), and timing changes are expressed in terms of sensitivity instead of implicit time.

**Figure 4:**
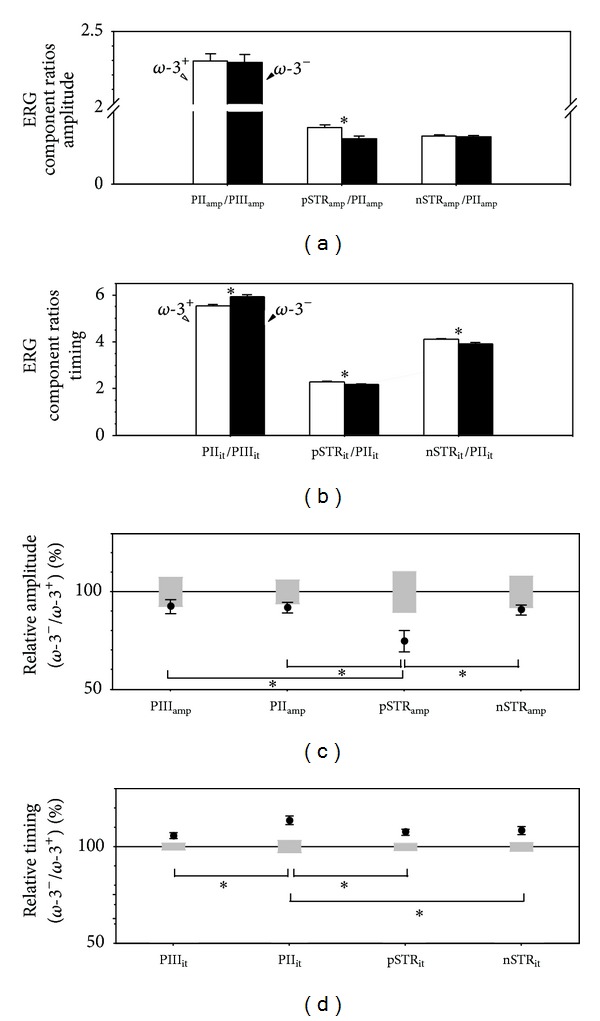
Previous approaches to determine “downstream effects”: ratio analysis ((a) and (b)) and percentage change relative to a normal cohort ((c) and (d)) of *ω*-3 dietary deficiency. Panels (c) and (d) are reproduced with permission from Nguyen et al. [28]. Asterisks (∗) indicate a statistically significant direct loss at the respective cell (*P* < 0.05). (a) Ratio analysis of amplitudes indicates no dietary change (*ω*-3^+^ white bars, *ω*-3^−^ black bars) in the PII_amp_/PIII_amp_ nor nSTR_amp_/PII_amp_ ratio but a significant decrease in the pSTR_amp_/PII_amp_ ratio. (b) Ratio analysis of implicit times indicates an increase in the PII_it_/PIII_it_ ratio and a decrease in the pSTR_it_/PII_it_ ratio and nSTR_it_/PII_it_ ratio. (c) Percentage change analysis relative to the average control value (±95% CI, grey shaded area) indicates that *ω*-3 deficiency (average ± SEM, filled circle) exhibits greater dysfunction in the pSTR than the PIII, PII, or nSTR (d) Percentage analysis also indicates a greater delay in timing in the PII than the PIII, pSTR, or nSTR. Thus when comparing the ratio and percentage change analysis with the gain analysis (Figure 3) differences can be noted. Ratio and percentage change analyses indicate that all inner retinal timing changes are direct effects (PII_it_, pSTR_it_, and nSTR_it_), whereas by taking into account gain between ERG components the timing delays of inner retinal components are attributable to the initial photoreceptoral change. Coincidentally, the amplitude changes are in agreement between the 3 analyses techniques.

Figures 4(a) and 4(b) show that dietarydeficiency did not change the ratio of PII_amp_/PIII_amp_ nor nSTR_amp_/PII_amp_ indicating that the PII_amp_ and nSTR_amp_ changes can be attributed to their upstream neuronal generators which in this case is due to photoreceptor changes induced by diet. In contrast, there is a significant change in pSTR_amp_/PII_amp_ ratio (∗ symbol) indicating a specific loss in the pSTR generator, the ganglion cells. Ratio analysis indicates that all timing changes cannot be fully accounted for by their upstream neuronal generators (Figure 4(b)).

Using percentage change analysis by expressing amplitude of treated eyes to control eyes (Figure 4(c)) indicates that the pSTR amplitude is more affected than the other amplitudes (∗ symbol), indicating a direct effect at the ganglion cell. In terms of timing, this approach (Figure 4(d)) indicates that the PII delay is greater than the other implicit times assayed (∗ symbol), suggesting direct effects at the PII, pSTR, and nSTR generators. Thus the percentage change approach is in agreement with the component ratio analysis, which is not surprising as they both assume a unity gain between ERG components.

More importantly, however, the results of these approaches differ from the conclusions made using the gain analysis (Figure 3). Ratio and percentage change analyses indicate that all inner retinal timing changes have an overlay of direct effects (PII_it_, pSTR_it_, and nSTR_it_), whereas by taking into account the normal gains between successive ERG components, the timing delays of inner retinal components can be attributed to the initial photoreceptor change. Coincidentally, the amplitude changes are in agreement between the three analytical techniques.

### 3.4. Application to Outer and Inner Retinal Dysfunctions

**Figure 5:**
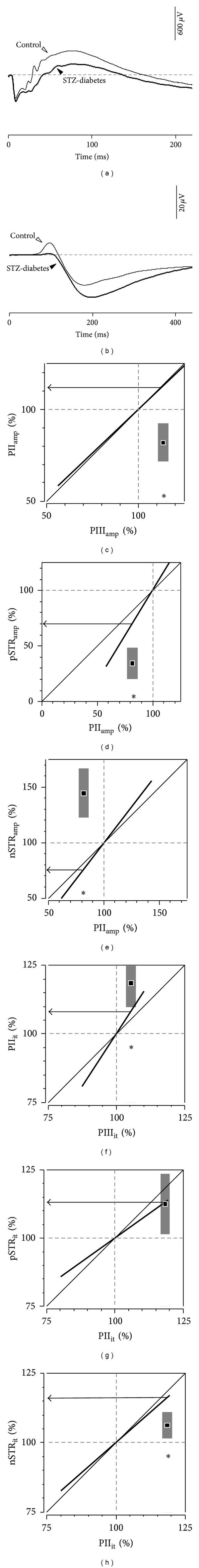
Application of ERG gain analysis to ERGs recorded from STZ-diabetic rats. Averaged group ERG waveforms showing the effect of diabetes on the (a) rod-isolated a-b wave complex and the (b) STR. (c) The thick line shows the ERG gain slope defined from the control group. Thin diagonal line shows the unity relationship. For a given reduction in input (along the *x*-axis) the predicted downstream change is given by the arrow (along the *y*-axis). This prediction can be compared to the measured change induced by the treatment (filled symbol) along with the 95% confidence limits (grey bar). This shows that PII loss cannot be due to photoreceptoral dysfunction. (d) Likewise, pSTR loss is greater than the PII deficits. (e) nSTR increased amplitude is not downstream of PII. (f) The delay in the PII is greater than that predicted from the PIII. (g) Delay in the pSTR can be accounted for by the PII delay. (h) nSTR delay is less than that expected from the PII delay. Asterisks (∗) denote significance (*P* < 0.05).

Figure 5 illustrates the application of the gain analysis to a separate cohort of animals. Figures 5(a) and 5(b) show representative control and diabetic waveforms. The most prominent diabetic changes evident in the waveforms are b-wave, OP (Figure 5(a)), and pSTR losses, as well as an increase in nSTR amplitude and delay (Figure 5(b)). From this, it is not clear whether the PII changes can account for the inner retinal losses. Our gain analysis shows that the measured PII (Figure 5(c)) and pSTR (Figure 5(d)) amplitude losses were both greater than what would be predicted from a normal gain, indicating a direct diabetes effect on these components. More specifically, the measured confidence limits for diabetes induced PII loss (−7.6% to −28.7%, grey box, Figure 5(c)) did not encompass the predicted PII increase downstream of the larger PIII (+12.7%, arrow). Similarly, the measured confidence limits for pSTR dysfunction (−51.3% to −80.1%, grey box in Figure 5(d)) were greater than the predicted loss (−29.7%, arrow) downstream of the PII change. The measured nSTR increase (95% confidence limits, +22.0% to +66.8%, grey box Figure 5(e)) was paradoxical given that a normal gain from the PII predicted a −23.5% loss (arrow). Thus, the measured nSTR changes due to diabetes cannot be accounted for by a normal gain relationship. 

In terms of timing (Figure 5(f)), the PII delay (+9.5% to +27.4%) was greater than that predicted (+8.1%), whereas the pSTR delay (+1.3% to +23.6%, Figure 5(g)) was consistent with the predicted delay (+13.1%). The nSTR delay (+1.4% to +11.2%, Figure 5(h)) was less than predicted (+16.2%). 

## 4. Discussion

### 4.1. Gain Relationships

This study defines the gains for different generators of the ERG for the first time in rodents. The PIII_amp_ to PII_amp_ gain relationship is unity (Table 1; 0.95 ± 0.04). This is in agreement with previous studies in humans [17, 18] and indicates that changes in PIII_amp_  are proportionately reflected in the  PII_amp_. In contrast, the PII_amp_ to STR_amp_ gains indicate amplification such that greater STR attenuation occurs for any given reduction in PII amplitude. That the gain is not unity at these third-order neurons may not be surprising, given the lateral communication, inhibitory feedback [22–24] and further convergence of signals [2, 25] that occurs at the inner plexiform layer. A more complex processing relationship at the third-order neurons is supported by the seminal studies conducted by Naka and colleagues [61–64] and in more recent studies [65]. These studies injected Gaussian white-noise currents into outer retinal neurons and measured the output profile from inner retinal generators. The authors found that photoreceptor stimulation yielded a linear bipolar cell output, whereas the ganglion cells responded in a nonlinear manner [61–65]. This is consistent with our findings of unity ERG gain between photoreceptors and bipolar cells but nonunity between bipolar and ganglion cells.

In terms of timing the gain relationship suggests that PIII_it_ delays are exaggerated at the  PII_it_, whereas PII_it_ delays are less prominent at the STR_it_. It is possible that the compression of delays from PII_it_ to STR_it_ may arise from inner retinal inhibitory feedback at this level, which is known to produce more transient cellular responses [23, 66–68]. As the gain relationship for timing characteristics differs from the gain relationships for amplitude, it is important to evaluate these separately.

### 4.2. Application to Photoreceptor Dysfunction and Its Effect Downstream

The predilection of *ω*-3 to affect photoreceptors is wellestablished, as photoreceptor outersegment has the highest docosahexanoic acid (DHA) content of any cell in the body [53, 54]. Indeed, a timing delay in the PIII (or a-wave) is the most common effect of *ω*-3 fatty acid deficiency found in a number of species including rats [55, 56]. Our laboratory has recently reported inner retinal deficits in rats fed *ω*-3 deficient diets [28], but it is not clear whether these losses arise from downstream of the known photoreceptor changes or involve direct dysfunction of retinal ganglion cells. A reanalysis of this data (with permission) using gain methods illustrates that all inner retinal timing changes are downstream of *ω*-3 deficiency-induced photoreceptor delays that in turn reflect reductions in photoreceptor sensitivity and  *t*_*d*_  delays. Thus, the apparently greater relative PII implicit time delay when compared with other ERG components does not indicate that rod bipolar cells are directly affected by *ω*-3 deficiency. Previous analytical approaches have led us to erroneously conclude this to be the case (Figure 4). Specifically, using a ratio analysis [19, 69] results in a PII/PIII timing increase (Figure 4(b)), and using the percentage change analysis [20, 21] gives greater delays in PII than PIII implicit time (Figure 4(d)). Both of these approaches falsely indicate a direct deficit at the PII level as they presume a unity relationship for implicit times between these processing stages. The gain analysis conducted in this study indicates that this is not the case (Figure 2). Indeed, when applied to *ω*-3 deficiency (Figure 3) the gain analysis shows that the greater PII timing delay can be wholly attributed to the PIII delay (Figure 3(f)). Similarly the smaller percentage STR delay (Figures 3(g) and 3(h)) can be attributed to a timing gain between the PII and STR of less than unity.

In terms of amplitude, the PII and nSTR changes can be attributed to downstream effects of reduced PIII input. However, the measured pSTR loss exceeds what would be expected from reduced outer retinal input, indicating a direct effect at the proximal retina (Figure 3(d)). As the pSTR is generated by ganglion cells in rats [16], we interpret this outcome as indicative of a direct effect of *ω*-3 deficiency on the retinal ganglion cells. Coincidentally, in this case the same conclusions would have been reached had the ratio (Figure 4(a)) or percentage change (Figure 4(c)) analysis been applied.

The finding for a preferential effect on photoreceptors and ganglion cells in *ω*-3 deficiency is in accordance with the major sites of *ω*-3 fatty acid incorporation in the retina as indicated by uptake of radio-labelled docoahexaenoic acid (^3^H-22 : 6). Systemic injections of ^3^H-22 : 6 result in pronounced labelling of photoreceptors and moderate labelling of the nerve fiber layer in the presence of minor labelling in the inner nuclear layer [70]. This labelling pattern suggests that both photoreceptors and ganglion cells are particularly dependent on *ω*-3 fatty acids, consistent with our noted dysfunction. 

### 4.3. Application to Outer and Inner Retinal Dysfunctions

 Retinal dysfunction in STZ-induced diabetes has been described extensively. The earliest changes appear to be specific to the inner retina [57–60]. However, it is not clear if these inner retinal deficits are due to reduced outer retinal input. Our analysis, shows that the PII (Figure 5(c)) and pSTR (Figure 5(d)) amplitude losses were greater than predicted from normal gains, indicating a direct diabetes effect on these components. The decreased PII amplitude and delay in PII timing are in accordance with other studies in diabetic rats [26, 71–73]. That diabetes had a direct effect on the PII is consistent with bipolar cell recordings from diabetic rat eyes showing reduced sensitivity to neurotransmitter application [74]. The pSTR amplitude decrease and nSTR amplitude increase (both also delayed) following diabetes support previous findings for similar changes from our laboratory [58]. The direct effect of diabetes on ganglion cells is supported by structural changes found in these rodent optic nerves, more specifically reduced fascicle area and increased proportion of the nerve taken up by blood vessels and connective tissue [57]. This is also consistent with increased apoptosis observed in the ganglion cell layer following STZ treatment in rats [59, 75] and mice [76] and in human sufferers of diabetes [60]. It is also consistent with the clinical observation that diabetic patients have a thinned retinal nerve fibre layer [77].

### 4.4. Assumptions of ERG Gain Analysis

It is clear that, for the same set of recording conditions, the gain relationships established are easily applicable to different sets of data. This approach provides a more complete picture as to the site of neuronal dysfunction across components of the full-field ERG. However, it is important to note that this analysis makes the following assumptions.

#### 4.4.1. A “Direct” Effect May Arise from Several Conditions

The cause of loss to postreceptoral ERG components can be “downstream” or “direct”. We employ the term “direct” to describe a number of possible scenarios. Firstly, the treatment may injure the postreceptoral neuron; secondly, it might impair neurotransmission between cells; thirdly, it might impair support (glia) or lateral cells involved in signal processing (e.g., amacrine cells). 

#### 4.4.2. Model Predicts Worst-Case Scenario

Due to the convergent characteristics of the rod pathway [2] different patterns of outer retinal loss will translate to inner retinal dysfunction in different ways. More specifically, Hood et al. [17, 78] simulated the disparate effects that homogeneous (photoreceptors damaged uniformly) and heterogeneous (photoreceptors affected unevenly, e.g., every second photoreceptor) photoreceptoral losses would have on bipolar cell ERG components. A homogeneous outer retinal loss produces a larger effect on the inner retinal responses [78]. A homogeneous loss has been assumed in the analysis developed in this paper and is reflected in the way in which the “gains” were determined. Whilst heterogeneous photoreceptoral losses may produce a different set of “gains”, the current approach is robust for the correct identification of inner retinal injury.

#### 4.4.3. Gain Relationships Assumed to Be Linear

We assume that the relationship between ERG components is linear, with slope being allowed to vary. In reality, gain relationships are likely to be more complex, but for the following reasons linearity is assumed in this analysis. Firstly, it is the simplest approximation to model our data, and the reduction in the degrees of freedom associated with a nonlinear function was not supported statistically. Indeed, even if the nonlinear neuronal generator relationships (as indicated by Naka and Colleagues [61–65] for bipolar to ganglion cells) manifest in ERG gains, the linear approach provides a close approximation over the short ranges used in our model (~±50%). Given most diseases produce mild to moderate deficiencies in the full-field ERG that fall within the range shown in this study, a linear assumption is likely to be appropriate. Secondly, a linear relationship has been shown to be appropriate for the gain relationship between the PIII and PII amplitudes [17, 18]. Thirdly, in eyecup preparations ganglion cells exhibited a linear rise in spiking rate with increasing input current once a graded membrane potential threshold is reached [79]. 

#### 4.4.4. Gain Relationships Only Applicable under Conditions Utilised during Recording

 As communication characteristics between neurons alter with adaptation [32–40] and degree of saturation [13, 30, 31] the conditions under which they are applied must be comparable to the conditions under which the gain relationships are derived. In the gain relationships defined in this paper this would be applicable only to dark-adapted, saturated responses of the rod pathway. 

## 5. Conclusions

The serial processing nature of the retina frustrates interpretation of inner retinal ERG dysfunction. Conventional ERG analyses that fail to account for “gains” between ERG components generated serial to each other will produce erroneous conclusions in identifying specific neural dysfunction. In this paper we determine the normal ERG gain relationships between saturated ERG components arising from the through rod pathway in rat, by correlating amplitudes and timings in a control group 

Our methodology has allowed us to establish that the gain between PIII and PII amplitudes is unity, which differs from the PII to the STR gains (p- and n-STR amplitudes). Indeed, the steeper gain slopes for both pSTR and nSTR amplitudes indicate that a given PII deficit will lead to a greater attenuation of STR amplitude. Furthermore, we establish that the implicit time gain slopes for the various ERG components are not unity. There is an exaggeration of delays between the PIII to PII and a compression from PII to STR components. As our gains have been scaled and normalised, they should be applicable to rod-generated signals collected in other labs. Should stimulus conditions differ, our approach can be applied by analysing amplitude and timing relationships in a control cohort under the test conditions utilised. What is important in such analysis is that some consideration be given to the gain relationship between components when interpreting ERG changes. 

We propose that the following criteria be observed in applying gain analysis to cohorts of animals form other labs.

1. ERG data collection:
  - dark adapted rod-ERG components need to be utilized,
  - saturated ERG components need to be employed.
2. ERG parameters:
  - achieved at a minimum of two luminous energies:
    - bright flash: 1.4 to 2.2 log cd·s·m^−2^ (paired flash for rod isolation),
    - dim flash: −5.5 to −4.8 log cd·s·m^−2^;
  - amplitude and implicit time derived from:
    - photoreceptor (PIII, bright flash),
    - bipolar cell (PII, paired flash using bright energy),
    - ganglion cell (pSTR, dim flash),
3. express treated ERG parameters as a percentage of control,
4. compare these data to the gains in Table 1 (Figures 3(c)–3(h)):
  - if the data has 95% CI that overlaps with ERG gain → downstream effect,
  - if the data has 95% CI that doesnot overlap with ERG gain → direct effect.

An alternative would be to utilise your own control cohort of animals to determine your strain/species gains within your experimental configuration and compare these against your treated cohort.

## Figures and Tables

**Table 1a:** (a)

Amplitude gains	Average	SEM	Upper 97.5% CL	Lower 97.5% CL
PIII_amp_ to PII_amp_	0.95	0.04	1.07	0.73
PII_amp_ to pSTR_amp_*	1.64	0.15	2.05	1.23
PII_amp_ to nSTR_amp_*	1.30	0.09	1.56	1.03

**Table 1b:** (b)

Timing gains	Average	SEM	Upper 97.5% CL	Lower 97.5% CL
PIII_it_ to PII_it_*	1.54	0.12	1.88	1.19
PII_it_ to pSTR_it_^#^	0.71	0.05	0.85	0.56
PII_it_ to nSTR_it_^#^	0.88	0.03	0.97	0.78

The average, SEM, and 95% confidence limits for the ERG regressions established from 5 trials in Figure 3. Amplitude (a) and timing (b) ERG gains whose 95% confidence limits encompass 1 indicate an ERG gain of unity. Those that are significantly greater than 1 indicate an amplification in gain (*) and significantly less than 1 a compression in gain (^#^). This indicates that the PIII_amp_ to PII_amp_ has a gain which encompasses unity whereas there is an amplification of gain between the PII_amp_ to pSTR_amp_ and the PII_amp_ to nSTR_amp_. In terms of timing, there is an amplification of gain between the PIII_it_ to PII_it_ in contrast to a compression of gain between the PII_it_ to pSTR_it_ and the PII_it_ to nSTR_it_.
